# Somatization symptoms—prevalence and risk, stress and resilience factors among medical and dental students at a mid-sized German university

**DOI:** 10.7717/peerj.13803

**Published:** 2022-08-19

**Authors:** Oskar Feussner, Carolin Rehnisch, Nadja Rabkow, Stefan Watzke

**Affiliations:** Polyclinic for Psychiatry, Psychotherapy and Psychosomatics, University Hospital Martin-Luther-University Halle-Wittenberg, Halle (Saale), Lower Saxony, Germany

**Keywords:** Medical students, Dental students, Somatization disorder, Somatoform disorder, Risk factor, Protective factor

## Abstract

**Objective:**

Previous studies have shown that an increased prevalence of mental illness can be found among medical and dental students. Among these, somatization symptoms are severely understudied. The present study examined the prevalence of somatization symptoms in a subpopulation of medical and dental students and aimed at finding associated risk and resilience factors.

**Methods:**

A cross-sectional survey was conducted using a self-reporting questionnaire, including the SOMS-2, the Becks-Depression-Inventory-II (BDI-II), the NEO-Five-Factor-Inventory, and a questionnaire on socio-demographics for possible risk and resilience factors. A total of 271 medical and dental students of a mid-sized German university completed the questionnaire.

**Results:**

The Somatization index yielded a mean of 9.12 symptoms for the total sample, which is 1.2 SD higher than the reported norm. A total of 50.7% of the medical students and 63.6% of the dental students transcend a critical somatization score. Significant positive associations for eight general risk factors, four university related stress factors, and a significant negative association for seven resilience factors were found.

**Conclusion:**

Medical and even more dental students at the studied university showed a high burden of somatoform complaints. Also, factors were found that could be of etiological relevance and others that could be used to enhance resilience. Both could present an opportunity for the prevention of somatization disorders but prospective and multicenter studies with an aged-matched comparison group are needed to obtain a more accurate overview.

## Introduction

Poor mental health and an increased prevalence of mental illness among medical and dental students has been repeatedly demonstrated worldwide (Dyrbye, Thomas & Shanafelt, 2006). Among these mental illnesses depression and anxiety disorders are of high prevalence and have received much scholarly attention. Etiologically, the high psychological pressure and the associated stress that medical students are exposed to as a consequence of a demanding curriculum, frequent examinations, a competitive environment, and the fear of clinical encounters are assumed to serve a critical role in the development of mental illnesses (Dyrbye, Thomas & Shanafelt, 2005; Yusoff et al., 2013). Another such mental illness that is believed to be triggered by stress are somatization disorders. Somatoform disorders (or “somatic symptom disorder” as in DSM-V (American Psychiatric Association, 2013)) are a group of mental disorders which are characterized by distressing somatic complaints for which no or no sufficient organic explanation can be found. In comparison to depression and anxiety disorders, somatization disorders among medical and dental students are severely understudied, even though they can be expected to be equally prevalent and debilitating (Quek et al., 2019).

The prevalence of somatoform disorders is substantial, as shown by a recent meta-analysis which found a prevalence of 26.2–34.8% of somatoform disorders in patients of general practitioners (Haller et al., 2015). The significance of somatoform disorders is exacerbated by the fact that somatizing patients have a disproportionately higher use of medical services with approximately twice the annual health care costs compared to non-somatizing patients. Even after adjusting for medical and psychiatrist comorbidity, estimated medical care cost were $256 billion per year in the United States for somatization alone, a value that places an immense burden on the health care system (Barsky, Orav & Bates, 2005). For the individual, somatization can lead to serious functional impairments, reduction of quality of life and work participation (Rask et al., 2015); in some cases to a greater extent than with well-defined medical diseases such as multiple sclerosis and rheumatoid arthritis (Rask et al., 2015; Joustra et al., 2015; Carson et al., 2011). In stark contrast to the significant relevance is the uncertainty about the classification and diagnostics of somatoform disorders, making them “one of the most controversial and challenging areas of modern psychiatry” (Creed, 2006; Zahid et al., 2016).

While the etiology of somatization disorders has not yet been conclusively determined, several possible risk factors have been identified. These include female gender (Jacobi et al., 2004), low socioeconomic status  (Creed & Barsky, 2004), financial worries (Wege et al., 2016), substance use (Hasin & Katz, 2007), neuroticism (Russo et al., 1997) and a frequent co-occurrence with other mental disorders, especially depression.

Further risk factors are psychological strain and stressful live events  (Chrousos, 2009; Tak & Rosmalen, 2010; Nater, Fischer & Ehlert, 2011). In particular, this observation was replicated in medical students, where not only a high frequency of somatization but a general correlation between academic stress and somatization and an increase in both stress and somatization from baseline to pre-exam period was found  (Brambila-Tapia, 2020; Kleine-Borgmann et al., 2021). Therefore, in our study we also assessed possible university-related stress factors like “competition among students”, “shortage of time” and “mental overload” to evaluate their impact in respect to somatization. The stress-related somatization among medical students may also be exacerbated by the fact that medical students acquire a great amount of clinical knowledge during their studies and “that this knowledge affects symptom perception via the expectations and illness beliefs (‘schemata’) that arise from it, leading to ‘selective attention’ to specific bodily sensations and areas” (Waterman & Weinman, 2014). Indeed, studies from different countries showed an increased prevalence of somatization, somatization syndrome, and the number of psychosomatic complaints among medical students compared to the general population (Brambila-Tapia, 2020; Carson et al., 2000; Adhikari et al., 2017).

To explore the question of whether somatization affects medical students in particular or other students as well, we also surveyed dental students, who are also subject to stressful studies. To the best of our knowledge, there is only one South Korean study showing a positive correlation between somatization symptoms and stress of clinical practice in dental students (Hong et al., 2009). Alzahem et al. (2011) report in their systematic literature review that dental students are subjected to substantially increased levels of distress during their studies. Furthermore, Humphris et al. (2002) found that not only the scores of psychological distress among dental students are comparable to those of medical students, but that the level of emotional exhaustion is even higher. The main reasons cited for this are the amount of assigned class work, fear of failing and/or being unable to catch up if getting behind, peer competition, academic overload, completing clinical requirements, responsibility for comprehensive patient care, performance pressure, shortage of allocated time and financial problems. In addition, an elevated prevalence of depression, anxiety disorders, and burnout has been identified among dental students in a number of countries around the world (Bathla et al., 2015; Basudan, Binanzan & Alhassan, 2017; Arrieta-Vergara et al., 2019). Studies comparing them to medical students showed an even higher incidence of anxiety, depression, burnout, depersonalization, exhaustion and hazardous drinking behavior among dental students  (Prinz et al., 2012; Newbury-Birch, Lowry & Kamali, 2002). Based on these findings on stress and mental health issues among dental students, we suspect that their burden of somatoform complaints is at least as severe as in medical students, if not worse.

With this study we seek to answer the question whether medical and dental students are particularly burdened by somatization disorders and whether they differ between each other in the prevalence and expression of individual risk and resilience factors despite similar courses of study. Further, we seek to enhance the understanding regarding the etiology of somatoform disorders, to identify potential risk and resilience factors and to offer abilities to establish targeted prevention and education regarding somatoform disorders.

## Material and Methods

### Design

A positive vote from the ethics committee of the Martin-Luther-University Halle-Wittenberg was obtained in March 2019 (Processing number: 2017-138). Before handing out the survey form, the students were informed about the voluntariness and the purpose of the study and declared their consent by participation.

For data collection, we conducted a cross-sectional survey using a self-reporting questionnaire, including the Screening for Somatoform disorders (SOMS-2-) questionnaire, the Becks-Depression-Inventory-II (BDI-II) and a questionnaire on socio-demographics. Participants did not receive any financial compensation.

For medical students the questionnaire was handed out in March 2019 during a mandatory course in the semester break where it was possible to fill in the questionnaire immediately but also to be collected later. Data collection in dental students was carried out in April and May during lectures with enough time to complete the questionnaire on site.

To ensure the anonymity of sensitive personal data, student names were anonymized by using pseudonyms that did not allow any indication of the actual name. The completed surveys were anonymously collected in a box.

### Participants

A total of *n* = 271 students of the medical faculty of a medium sized public university in Germany participated. Of those, *n* = 129 (47.6%) were students of dental medicine and *n* = 142 (52.4%) were students of human medicine.

All medical students surveyed were in the 5th semester, which is the first clinical semester after two years of preclinical education and after the first state examination. Due to the small number of dental students enrolled in each academic year, we distributed the questionnaire in all of the five academic years. Therefore, two preclinical and three clinical academic years were examined.

Of *n* = 231 medical students that were asked to participate, *N* = 142 completed the questionnaires, representing 61.5% of the students in the included cohort. Participating dental students ( *N* = 129) represented 68,6% of all *n* = 188 dental students enrolled at time of the study. The response rate was 82% of all *n* = 157 that were eligible for the study. The selection process is illustrated in supplemental Fig. 1.

**Figure 1:**
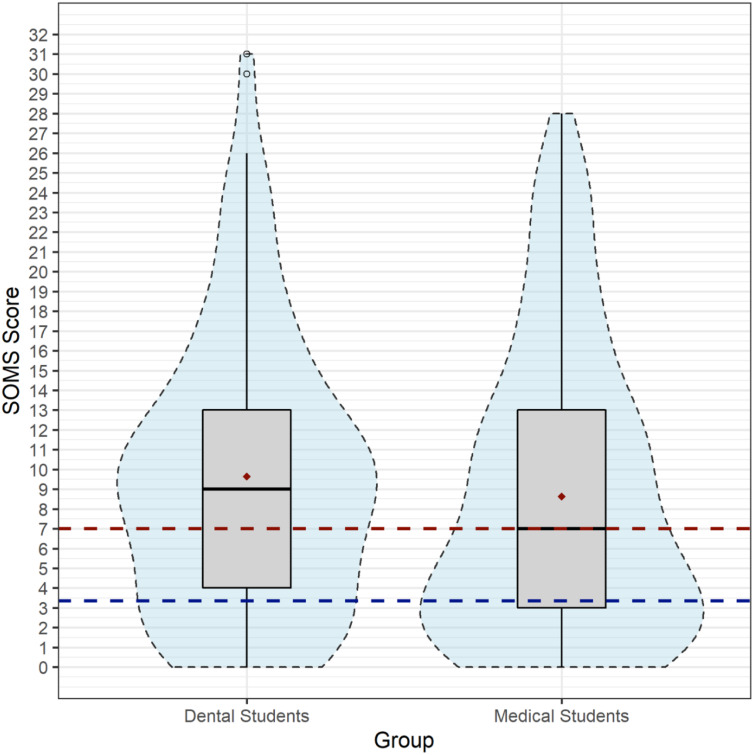
Distribution of symptom frequencies of the somatization index among dental and medical students. The box plot shows the distribution of symptom frequencies of the somatization index among dental and medical students. Furthermore, the mean, the median, the reference value of the general population (blue line = 3.35), and the cut-off value of seven (red line) are shown.

Despite the partially different semesters between the surveyed dental and human medicine students, the two groups did not differ significantly in age (F[*df* = 1]=1.51; *p* = .211). Dentistry students had a mean age of 23.79 (*SD* = 4.21) years, medical students had a mean age of 23.21 years (*SD* = 3.44). Biological sex was female for 66.7% of the dentistry students and 70.4% for the medical students (X^2^[*df* = 1]=.443; *p* = .506). The sampling error for medical students was 5.1% (CI 95%), for dentistry 4.8% and the overall sampling error was 3.5%.

### Instruments

Participants completed the following four questionnaires.

### Sociodemographic questionnaire

The first questionnaire included questions about socio-demographic data, some of which represent possible risk and resilience factors. These are presented separately below. The questionnaire included: age, gender, region of origin, socioeconomic and relationship status, religiousness, occupation before the studies, reasons for the election of the study course, number of siblings and children, parents highest educational and vocational degree as well as parents’ current occupational activity. In addition, information was requested about family history of mental illness, death of a parent, information regarding alcohol consumption and use of other substances, own psychiatric illness, relationship issues, family problems, and stressful study-related events such as high workload and upcoming events.

Furthermore, emotional support, hobbies (*e.g.*, playing an instrument, physical activity), specific relaxation techniques, time for family, friends and hobbies were surveyed as well as the satisfaction with the own body, diet, studies, partnership, friendship, family relationship and hobbies.

### Becks depression inventory-II

The second questionnaire was the BDI-II, which is considered to be an indicator of the presence and severity of depression. It is a self-report questionnaire in which individuals are asked 21 questions about depressive symptoms in the past two weeks. Each item is answered on a four-point Likert scale (0–3), resulting in a maximum score of 63.

A score above 14 can be interpreted as a mild depression, a score above 19 as moderate depression and above 28 as a severe depression. Studies have repeatedly demonstrated the high validity and reliability of the test (Arnau et al., 2001).

### The NEO-five-factor-inventory

In third place, we used the German version of the NEO-Five-Factor-Inventory (NEO-FFI) (Costa & McCrae, 2008), a standardized tool, representing the gold standard of personality self-assessment. For each scale, there are 12 items for which the participant had to choose between strong rejection (0/5) and strong approval (5/5) on a Likert scale. For this study, we only measured Neuroticism, one of the five basic personality factors.

### Screening for Somatoform disorders (SOMS-2)

To examine the prevalence of somatoform symptoms and disorders and enable the broadest possible comparability of our data we used the SOMS-questionnaire as the 4th questionnaire. The SOMS is a self-rating questionnaire designed for the detection of physical complaints without any structural pathology explaining the symptom, and associated distress within the past two years. It consists of 48 items for men and 52 items for woman with the response options “yes/no”, considering the International Classification of Disease 10th Revision (ICD-10) as well as the DSM-IV criterions/-somatization disorder symptom list. By summation of the individual items, there are three different possibilities of evaluation/interpretation: 1. DSM-IV Somatization disorder; 2. ICD-10 Somatization disorder and 3. ICD-10 somatoform autonomic dysfunction. Summing up all complaints yields a fourth integrated interpretation possibility: the “Beschwerdeindex Somatisierung” (= somatization index). Standard values are given in form of percentile ranks and refer to the general population. The frequency of the different symptoms is between 1% for blindness and 73% for back pain. For the SOMS-somatization index, Rief and Hiller found that “a cut-off of seven or more symptoms yielded the best discrimination between low and high disability” (Rief & Hiller, 1999). Therefore, a cut-off at seven was applied in our calculations.

### General risk factors, university-related stress factors and resilience factors

A total of 12 general risk factors (Creed & Barsky, 2004; Wege et al., 2016; Hasin & Katz, 2007; Russo et al., 1997; Franck et al., 2022; Fritz, Fritsch & Hagino, 1997; Lieb, Meinlschmidt & Araya, 2007; Lieb et al., 2002; John et al., 2004), six university-related stress factors and eight resilience factors that, according to existing literature are assumed to be associated with somatoform disorders were evaluated in the questionnaires. The following sections briefly elaborate on the factors mentioned.

### General risk factors

1. “Positive family history of psychiatric illness” was rated as present if one or more first- or second-degree family members (siblings or (grand-)parents) were in treatment because of a mental disorder other than Alzheimer’s disease or another dementia.
2. The risk factor “Own psychiatric illness” was defined to be present if the participant was or still is in treatment because of a mental disorder.
3. The BDI-II-Score was defined to be a risk factor with a cut-off at 13 points.
4. Female gender was assessed as both biological sex and as the person’s gender identity.
5. Not having a partner or being in a relationship shorter than three months was considered a risk factor.
6. The risk factor “low socioeconomic status” was defined to be present if the participant stated both parents’ educational and vocational career as “unskilled”, “without graduation” or “lower secondary education”.
7. “Loss or separation of a parent in childhood” has been used to determine if the participant had lost a parent by divorce or death.
8. If the participants answered the question “Do you have sufficient financial funds available?” with “sometimes too little”, “often too little” and “I am mostly under great financial pressure”, the risk factor “financial burden” was considered to be present.
9. Drinking eight drinks as a male or six drinks as a female in one session more than once a month was defined as the risk factor “alcohol consumption”.
10. “Use of drugs or medication” was rated present as a risk factor if the respondent indicated their consumption of medication or drugs to improve their concentration, for sedation or for sleep.
11. A daily cigarette consumption of one cigarette or more was defined as a positive determination of the risk factor “nicotine abuse”.
12. Neuroticism as a risk factor was operationalized if the individual’s sum score of the NEO-FFI subscale was higher than the mean + 1 SD of the representative norm

### Stress factors related to university content

In order to capture any possible stress factors related to university content, the students were asked: “Are there any things that currently make it hard for you to be happy with your study decision?”. In accordance with existing literature, participants could select one or more of the following predetermined responses, if applicable: “Uncertain prospects”, “Competition among students”, “Shortage of time”, “Excessive demands/mental overload”, “Pressure to perform” and “Loneliness”. These were determined as stress factors related to university content (Dyrbye, Thomas & Shanafelt, 2005; Hill, Goicochea & Merlo, 2018).

### Resilience factors

To further sharpen the understanding and create potential opportunities for intervention, we here surveyed resilience factors, including, for example, “emotional support” and “physical activity”. Both physical activity (Ströhle et al., 2007) and emotional support (Mak & Zane, 2004) have already been described as a protective effect in relation to somatoform disorders. The remaining factors were selected due to their demonstrated protective effect in relation to other mental disorders like depression, as their has yet been only limited research on resilience factors for somatoform disorders (Lolak et al., 2008; Bengel & Lyssenko, 2012; Psaltopoulou et al., 2013; Kindt et al., 2021).

The first resilience factor: “using of relaxation techniques (*e.g.*, PMR, yoga)” is rated present if the respondent answers “yes” to the question.

To fulfill criteria for the resilience factor “satisfaction with the study”, respondents needed to answer positively to three following questions: “Do you enjoy your studies?”, “How satisfied are you in overall with your studies?” and “From today’s perspective, would you decide to study medicine again?”.

“Religion” was included as a resilience factor if its importance in respondents’ daily lives was described as “extremely important” or “moderately important”.

Participants who answered “Do you have regular meals?” and “Do you pay attention to a healthy diet?” with “yes” or “mostly” met the requirements for the resilience factor “healthy diet”.

By answering the questions on “doing sports” with “more than two hours per week” and on “actively making music” with “more than one hour per week”, these resilience factors were rated present.

To include the resilience factors “sufficient emotional support” and “social contacts”, participants were asked “Do you experience sufficient emotional support?” and “How much time do you have available for friends, family, and partnership?”. Answering both questions with “more than enough” or “enough” was necessary for the resilience factors to be considered present.

### Data analysis

The Statistical Package for Social Sciences (SPSS 25.0) was used to carry out the statistical analysis. Descriptive statistics (M, SD or frequencies, percent) were used for the sociodemographic description of the sample. Normal distribution of metric variables were tested using Kolmogoroff-Smirnov-test. Depending on the outcome, group differences between medical and dentistry students were considered using *χ*2 and F-test statistics or non-parametric tests. The investigation of the prevalence of somatization symptoms and the description of the SOMS scores of the two groups resulted from descriptive statistics (M, SD) and determination of the absolute and relative frequencies of fulfillment of diagnostic criteria. To show potential connections between risk factors, stress factors, resilience factors, and the somatization index were tested by Pearson’s r or Spearman rank correlation (*r*_s_) in case of deviation from normal distribution respectively. Multiple stepwise regression analysis including all significant correlating risk and resilience factors were applied in order to identify the strongest risk and resilience factors. To address the problem of multicollinearity we used a stepwise forward model (pin < 0.05; pout > 0.10).

## Results

### Somatization index and prevalence for somatization disorder according to DSM-IV and ICD-10

As the primary outcome, the SOMS-somatization index yielded a mean of 9.12 symptoms (*SD* = 7.15; SEM = 0.43; 95% CI [−2.72–0.70]) for the total sample (*n* = 271). Dental students were found to have a higher mean score (*M* = 9.65, *SD* = 7.00; SEM 0.62) compared to medical students ((*n* = 142): *M* = 8.64, *SD* = 7.27; SEM = 0.61). Since somatization index was not normally distributed, group difference was tested using Mann–Whitney-U. The two study groups did not significantly differ (*U* = 8230; *p* = .149).

A critical somatization index of minimum 7 points was found in *n* = 154 subjects of the total sample (56.8%). Dental students showed a significantly higher frequency (*n* = 82, 63.6%) than medical students (*n* = 72, 50.7%; X^2^[*df* = 1]=4.558; *p* = .033).

Figure 1 visualizes the distribution of symptom frequencies of the somatization index among medical and dental students in a box plot. As can be seen in Fig. 1, dental students show a higher median (nine symptoms) and higher maximum scores. Furthermore, a significantly larger proportion exceeds the reference value of the general population and the cut-off value, with a concentration at 11 symptoms per individual. Medical students, on the other hand, show a larger clustering in the lower symptom ranges with a maximum at three symptoms.

*N* = 3 subjects of the total sample fulfilled the ICD-10 diagnostic criteria for somatoform disorders, which yields an ICD-10 diagnosis frequency of 1.1%. After applying criteria of DSM-IV, somatoform disorder was found in *n* = 7 subjects (2.6%) of the total sample, which included all of the ICD-cases. Age did not significantly correlate with SOMS-somatization index (*r* = 0.09).

### Risk and resilience factors

#### Somatization index related to general risk factors, university related risk factors and resilience factors

Table 1 shows the bivariate relationship between the SOMS-somatization index and risk and resilience factors, as well as the prevalence of risk and resilience factors within the two groups of students. In addition, the distribution differences of the variables (Chi-Square Test, *χ*2 test) are included in the last column.

**Table 1:** Bivariate relationship between risk/resilience factors and the somatization index, prevalence of risk/resilience factors within the two groups of students, and *χ*2 test on contingency between the variables ‘course of study’ and ‘risk/resilience factor’.

		Course of study		
	Somatization index *r*_*s*_	Medicine (%)	Dentistry (%)	*χ*^2^ (*df* = 1)
**Risk factor**				
Positive family history of psychiatric illness	0.10	37.3	38.8	0.043
Own psychiatric illness	0.22^**^	9.0	12.5	0.552
BDI-II >13	0.44^***^	22.8	40.2	9.23^**^
Female sex	0.27^***^	70.4	66.7	0.443
Being single	−0.14^*^	44.7	38.7	0.97
Low socioeconomic status	0.05	1.5	2.3	0.16
Loss of parent	0.12	17.9	12.6	1.00
Financial worries	0.17^*^	17.6	24.0	1.70
Alcohol consumption	−0.05 12.0	12.4	0.01
Drug abuse	0.23^***^	14.2	10.9	0.68
Nicotine abuse	0.18^**^	11.6	12.2	0.02
Neuroticism (>*M* + 1*SD*)	0.25^***^	9.0	21.9	5.10^*^
**Study related stress factors**				
Uncertain prospects	0.07	2.8	0.8	1.56
Competition among students	0.12^*^	9.2	9.3	0.00
Shortage of time	0.12^*^	62.7	64.3	0.08
Excessive demands / mental overload	0.20^***^	21.1	34.1	5.74*
Pressure to perform	0.24^***^	50.0	66.7	7.70^**^
Loneliness	0.07	13.4	13.2	0.00
**Resilience factor**				
Use of relaxation techniques	0.11	21.3	24.2	0.33
Study satisfaction	−0.18^**^	73.0	69.5	0.41
Religiousness	−0.04 26.9	17.8	2.17
Healthy diet	−0.06 65.5	47.3	9.13^**^
Actively making music	−0.04 27.7	22.4	0.97
Sport	−0.16^**^	63.4	69.3	1.05
Emotional support	−0.24^***^	81.0	76.0	1.01
Time for social contacts	−0.15^*^	17.6	11.6	1.92

**Notes.**

*r*_*s*_ = Spearman rank correlation, *N* = 271, medical students *n* = 142, dentistry students *n* = 129, correlation *r* between Somatization index and BDI-II total score *r* = .54***

* *p* ≤ 0.05.

** *p* ≤ 0.01.

*** *p* ≤ 0.001.

On average, medical students reported *M* = 2.37 risk factors (*SD* = 1.40), whereas dentistry students reported *M* = 3.09 (*SD* = 1.83) risk factors (Mann–Whitney-*U* = 6973; *p* = 0.001). The number of individual risk factors was correlated with SOMS-2 somatization index with *r*_*s*_ = 0.474 (*p* < 0.001).

The sum of study related stress factors had a mean of 1.59 for medical students (*SD* = 1.24) and 1.88 (*SD* = 1.21 Mann–Whitney-*U* = 7906,5; *p* = 0.045) for dental students. The individual sum of stress factors correlated with SOMS-2 somatization index with *r*_*s*_ = 0.280 (*p* < 0.001).

The sum of resilience factors was *M* = 3.61 (*SD* = 1.48) in medical students and *M* = 3.36 (*SD* = 1.56) in dentistry students (Mann–Whitney-*U* = 8244, 5; *p* = 0.148). The correlation with SOMS-2 somatization index was *r*_*s*_ = − 0.207 (*p* < 0.001).

### Stepwise linear regression analysis

A stepwise linear regression (stepwise (*p*_*in*_ =0.05; *p*_*out*_ =0.10)) regarding all significantly correlating factors of risk, stress and resilience explained 22% of the variance in the somatization index (corr. *R*^2^) and included BDI-II score >13, own psychiatric illness and female sex.

A regression model regarding study related stress factors explained 8% of the variance in the SOMS-somatization index and included pressure to perform and excessive demands/mental overload.

Regarding resilience factors, regression showed a *R*^2^ = .08 and included emotional support, satisfaction with studies and sport.

## Discussion

Research on somatoform disorders among medical students is scarce, and on dental students, knowledge is almost nil. Furthermore, the majority of recent studies did not take the possible effect of risk and resilience factors for somatoform disorders among students into account. Therefore, the present study examined the prevalence of somatoform disorders in a subpopulation of medical and dental students and aimed to find associated risk and resilience factors.

### Medical and dental students seem to have a high burden of somatoform complaints

With an average of 8.64 for medical students and 9.65 for dental students, our study demonstrates a high burden of somatoform complaints in medical and dental students (see Fig. 1). According to the SOMS manual, a number of nine symptoms result in a percentile score of 83 for healthy individuals, representing the top end of the general population. Taking both groups together, 56.8% transcend the critical somatization index of 7 points. In contrast, participants representing the general population in the studies by Rief and colleagues as well as Hessel and colleagues reported a mean score of only 3.35 unexplained symptoms (Rief, Hessel & Braehler, 2001; Hessel, Geyer & Brähler, 2002).

As opposed to the high number of reported symptoms, only 1.4% of the medical and 0.8% of the dental students fulfilled the ICD-10 criteria for somatization disorder. The DSM-IV criteria for somatization disorder were met in 1.4% of the medical and 3.9% of the dental students. In comparison, Hessel and colleagues found that only 0.3% of their participants representing the general population met ICD-10 or DSM-IV criteria, respectively (Hessel, Geyer & Brähler, 2002). Other studies that have investigated somatization disorder in primary care patients show a point prevalence of 0.8% for DSM-IV and 4.7% for ICD-10 (Haller et al., 2015).

However, it should be noted that due to the highly heterogeneous diagnostic systems (ICD, DSM-III/-IV/-V), different forms of prevalence (point *vs.* 12-month- *vs.* lifetime prevalence), diverse study populations (population-based, primary care, secondary care) and differences in used measurement instruments, a general comparability of studies on somatoform disorders is challenging. Further research based on the same diagnostic systems and measurement instruments is required to enhance the comparability of findings.

Despite these limitations of general comparability, we have observed an unusually high number of complaints. This may be due to the high burden of psychological complaints in the sample we studied or because medical and dental students deal with somatic complaints and their consequences on a regular basis as part of their studies (Pukas et al., 2022; Rehnisch et al., 2021). Our findings of higher somatic symptom burden in medical and dental students indicate the great relevance. The importance is further highlighted by the fact that 32.8% of students surveyed reported taking medication due to the complaints. Even more troubling is the fact that 38.7% said the complaints had a significant impact on their well-being.

Comparing our results with the few studies that have used medical students as a study population, comparable results emerge. A previous study by Wege and colleagues found a significantly higher prevalence rate of somatization syndrome among female medical students and a higher sum score for psychosomatic complaints in medical students than in the general German population (4.1 *vs.* 1.8 for men and 6.8 *vs.* 3.2 for woman) (Wege et al., 2016). Further evidence of high burden can be found in the study by Brambila-Tapia (2020) who observed a prevalence of somatization in 66.59% of medical students. This is also underlined by the results of Adhikari et al. (2017), who observed moderate to severe somatic symptoms in 22.4%. In contrast, Campos et al. discovered a slightly lower rate of somatization among medical students in Portugal compared to the general population (Campos et al., 2021). Looking at DSM-IV somatization disorder, a comparable fraction of 3.7% of medical students meeting the criteria was found in the past (Zaki & Ibrahim, 2011).

The assumption that the challenging and stressful study is a decisive trigger for mental disorders is supported by the observation that the mental health of medical students at the beginning of the first year seems to be comparable to that of the general population and only deteriorates over the course of the following years in medical school  (Carson et al., 2000; Rosal et al., 1997).

To the best of our knowledge, no study has previously investigated the prevalence of somatization disorders among dental students. Our results suggest that dental students are even more affected than medical students. Aiming to account for the observed high prevalence and the differences between the two study programs, several risk and resilience factors are presented and discussed in the following.

### Of the twelve general risk factors eight showed a significant positive association

As can be seen in Table 1, we have not been able to demonstrate a significant positive association for the following risk factors reported in the literature: “Lower socioeconomic status”, “Positive family history of psychiatric illness”, “Loss of parents” and “Alcohol consumption”.

### BDI-II-Score of more than 13 points shows the highest association with the somatization index

However, there is also a substantial disparity between students of human and of dental medicine. Since the latter ones, in comparison to medical students, surpass the score of 13 in almost twice as many cases, their higher prevalence of depression might be one reason for the higher rate of somatization among dental students. This is supported by the findings of Prinz et al. (2012) as well as Newbury-Birch, Lowry & Kamali (2002), who observed higher levels of depression in dental students compared to medical students.

The general association of an elevated depression score with the somatization index is consisting with previous findings (Henningsen & Löwe, 2006; Mergl et al., 2007; Lieb, Meinlschmidt & Araya, 2007; Hanel et al., 2009). Mergl et al. (2007) showed that 44.55% of all patients with somatoform disorders met the diagnostic criteria for depression. This co-occurrence is so pronounced that some authors even assume a common identity and state: “Epidemiologically, the overlap of depression, pain and somatoform disorders continues to be the rule rather than the exception“ (Henningsen & Löwe, 2006). However, in the context of high comorbidities, it is important to consider that the diagnostic criteria and screening instrument may not be specific enough to distinguish between different pathologies (Mergl et al., 2007). The awareness of a common comorbidity is not only essential for therapy, but can also provide valuable knowledge about the etiology and thus improve the classification of mental disorders (Lieb, Meinlschmidt & Araya, 2007).

### Neuroticism showed the second highest association with the somatization index

Dental students score significantly higher, making it the only other “general risk factor” in which medical and dental students differ significantly. These findings are in accordance with literature that attributes a critical role to neuroticism in the development of somatization and medically unexplained symptoms (Russo et al., 1997; Costa & McCrae, 1987; Van Dijk et al., 2016). This connection is explained by the assumption that neuroticism represents a general vulnerability to psychological stress and, through stress intolerance, lowers the threshold for symptom perception (Deary, Chalder & Sharpe, 2007) through different mediating mechanisms (somatic sensitivity, selective attention, and negative reporting bias).

### The positive association with an own psychiatric illness confirms previous observations

Jacobi et al. (2004) found that 54.3% of the patients diagnosed with any somatoform disorder had at least one additional psychiatric diagnose (12-month prevalence). Also De Waal et al. (2004) showed that “Comorbidity of somatoform disorders and anxiety or depressive disorders was 3.3 times more likely than could have been expected by chance”. It is particularly alarming that patients with somatoform disorders have suicidal thoughts in 24% and have already attempted suicide in 17.6% (Wiborg et al., 2013).

Furthermore, Garcia-Campayo et al. (2007) as well as Eck et al. (2015) reported an increased comorbidity of somatoform disorders and medically unexplained symptoms to personality disorders and any substance use. In addition, patients with the comorbidity of a somatoform disorder and another psychiatric illness usually have a higher level of distress and functional impairment  (Jacobi et al., 2004; Hüsing et al., 2018).

### Female students score on average higher on the somatization index than their male counterparts

This finding is again in agreement with existing literature (Jacobi et al., 2004; Lieb et al., 2002; Schulte & Petermann, 2011).

### Being single may be regarded as a protecting factor

Regarding the civil status, the literature does not allow a clear statement. An association between somatization disease and unmarried status was observed in only half of the studies reviewed by Creed & Barsky (2004). The negative association we found between “being single” and the somatization index might therefore imply a protective role.

### Financial worries are a risk factor

Our observations are consistent with those of Wege and colleagues, who discovered a significant association between expected financial difficulties and psychosomatic complaints in medical students (Wege et al., 2016). These findings are further supported by the fact that a connection between financial worries and somatization symptoms has also been described among refugees (Junod Perron & Hudelson, 2006; Bäärnhielm & Ekblad, 2000). Furthermore, it has already been shown that financial worries can lead to depression and it can, therefore, be assumed that they represent a vulnerability factor  (Tsuchiya et al., 2020; Weissman, Russell & Mann, 2020).

### Other risk factors are drug abuse and smoking

Even though the negative association between our somatization index and alcohol consumption is not significant, we were able to demonstrate a significant association with drug abuse.

In their review on somatoform and substance use disorders, Hasin & Katz (2007) report partly contradictory results, but conclude that a connection between somatoform disorders and substance use disorder can be assumed. Yet, the authors object that many of the symptoms of substance use disorder overlap with possible symptoms of somatoform disorder.

John et al. (2004) show that individuals who currently smoke are more likely to have a somatoform disorder. This corresponds to the positive association we were able to demonstrate between nicotine abuse and the somatization index.

###  4 out of 6 analyzed stress factors related to university context contribute to somatoform disorders

While innumerable studies have demonstrated high psychological distress in medical students and linked it to mental issues like depression and anxiety disorder (Dyrbye, Thomas & Shanafelt, 2006; Dyrbye, Thomas & Shanafelt, 2005; Sreeramareddy et al., 2007), this research makes a definitive contribution to knowledge about somatization complaints. Of the six university-related risk factors examined, four show a significant positive association (presented in Table 1). While it has been shown consistently in recent decades that medical studies are very demanding, several studies have pointed out that the study of dentistry, with its partially similar curriculum, represents a comparable challenge (Alzahem et al., 2011; Elani, Kumar & Bedos, 2014). Especially with regard to the higher scores of dental students on the risk factors “Excessive demands/mental overload” and “Pressure to perform”, our research shows that dental students might even be more stressed during their studies. This observation is supported by a study of Birks, McKendree & Watt (2009).

### Only half of the analyzed resilience factors revealed a protective effect

Except for “use of relaxation techniques”, all resilience factors showed the hypothesized negative association with somatization severity. However, only half of them reached significance, indicating a protective effect. Hereby, our findings are consistent with previous observations that somatoform disorders are less likely to occur with regular physical activity and the presence of emotional support  (Ströhle et al., 2007; Mak & Zane, 2004).

### Limitations of this study and future research

The size of the study sample was well appropriate to reveal bivariate relationships and to compare dental to medical students. Also, multivariable procedures (regression analysis) were used to investigate the relationships of risk factors among themselves. However, it must be noted that the multiple regression analysis showed that only a 22% variance could be explained by the factors studied.

Furthermore, due to limited time and financial resources, we conducted a cross-sectional study, knowing that it does not allow inferences regarding the sequence of events and causality. For these very reasons, as well as the fact that this is a pilot study, we did not conduct standardized psychiatric interviews but used self-report-questionnaires. Therefore, we cannot make definite clinical diagnoses, as we cannot determine accordance rates of self-report-questionnaires with interview-focused diagnoses.

A limitation is the sample size which cannot be representative enough to draw conclusions for the population of German medical and dental students. However, since we can assume that, due to national regulations, structure and curriculum for the study of medicine and dentistry are very similar throughout Germany, the results are presumably comparable to students of other universities.

It should also be noted that we only surveyed medical students from the fifth semester and, in comparison, dental students from all semesters due to the smaller groups size in each semester. However, since the fifth semester represents approximately the middle of the study period and the two study groups did not differ significantly in age, it is questionable if this factor significantly restricts the interpretability of the results.

Despite these limitations, our study makes a valuable contribution to the existing literature and to the understanding of somatization. However, prospective and multicenter studies with an aged-matched comparison group are needed to obtain a more accurate overview of the severity of somatoform complaints among medicine and dental students. In addition, these studies are needed to answer how severe the burden is at the beginning of the study, how it changes in the course and whether it is still apparent in later working life. It would also be interesting to know how prevalence differs with the new criteria from DSM-V compared to DSM-IV and if students from other universities and courses are also affected.

## Conclusions

Our study reveals that medical students and even more so dental students are significantly affected by somatoform complaints. We were also able to demonstrate that factors such as mental illness (particularly depression), female gender, drug and nicotine abuse, and neuroticism were correlated with an increased symptom burden and are thus of etiological relevance to the development of somatization disorder. In addition, stress arising from the study course has a significant association with somatization. In contrast, there are factors like emotional support, physical activity, study satisfaction and sufficient time for social contacts which display a protective effect. A possible explanation for the higher expression of somatization symptoms among dental students is the fact that they displayed higher levels of neuroticism, more frequently exceeded a critical BDI score and that they were more often mentally overloaded and under pressure to perform during their studies. However, associations between somatization and the factors studied were moderate. This indicates a multivariate model of somatization with no single factor contributing solely.

Consequently, medical students and even more urgently dental students need to be educated at an early stage about the increased prevalence, possible risk factors, comorbidity and consequences of somatization. Furthermore, the knowledge thus acquired about the occurrence and handling of medically unexplained symptoms and somatoform disorders may enable tomorrow’s practitioners to recognize and deal more efficiently with medically unexplained symptoms and somatoform disorders, and hence enable them to reduce years of disability and financial costs in patients. However, educating students will not be sufficient. In addition to the education of the students, it is also important to reduce psychological/university-related distress. Possible approaches to stress reduction include mindfulness-based stress reduction (Rosenzweig et al., 2003), student-led stress management programs (Redwood & Pollak, 2007), time management programs  (Lincoln, Adamson & Covic, 2004), and promoting coping strategies (Wolf, 1994), among others. This could not only reduce university-related stress but also increase study satisfaction and create more time for social contacts, which in turn, has a protective effect, as shown above. It can also help to promote regular physical activity, as it provides a preventive effect towards somatoform disorders, as well (Ströhle et al., 2007).

To conclude, there are many approaches and possibilities to reduce the prevalence and improve the diagnosis of somatoform disorders. More attention and relevance need to be given to this topic in the future.

## Supplemental Information

10.7717/peerj.13803/supp-1 Data S1 Total responses of study participants to the survey questions

10.7717/peerj.13803/supp-2 Figure S1 Strobe flowchart to visualize selection process of participating medical and dental students The flowchart illustrates the selection process of participating medical students on the left and dental students on the right.

## Additional Information and Declarations

### Data Availability

The following information was supplied regarding data availability: The raw data of total responses of study participants to the survey questions is available in the Supplementary Files.
