# Supplementary figures and images for: Somatization symptoms—prevalence and risk, stress and resilience factors among medical and dental students at a mid-sized German university
Source: PeerJ. 2022 Aug 19;10:e13803. doi: 10.7717/peerj.13803 (PMC9394510; doi:10.7717/peerj.13803)

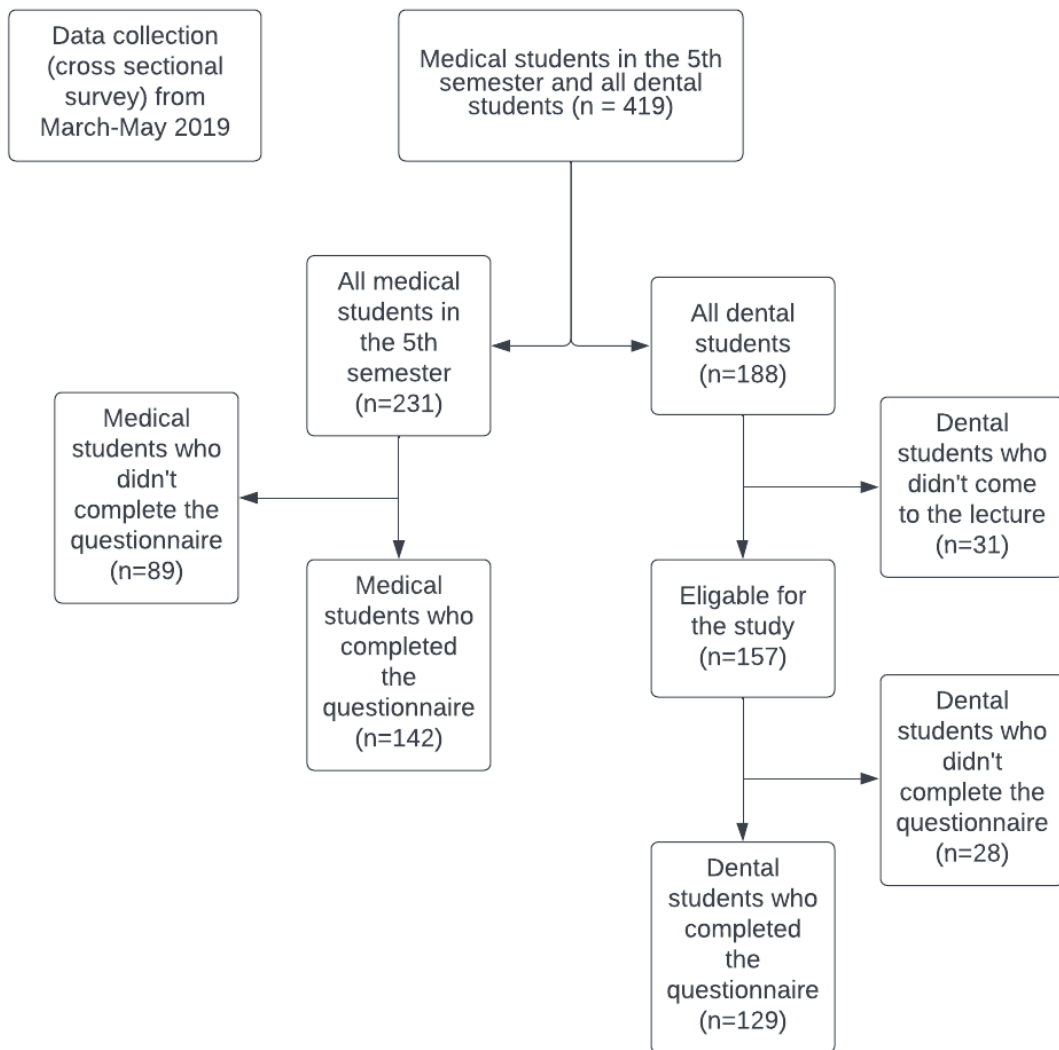

Supplement: Figure S1 — The flowchart illustrates the selection process of participating medical students on the left and dental students on the right. [file peerj-10-13803-s002.pdf]
